# Impact of metal oxide nanoparticles on in vitro DNA amplification

**DOI:** 10.7717/peerj.7228

**Published:** 2019-06-27

**Authors:** Chun-Hui Gao, Monika Mortimer, Ming Zhang, Patricia A. Holden, Peng Cai, Shan Wu, Yuexing Xin, Yichao Wu, Qiaoyun Huang

**Affiliations:** 1 State Key Laboratory of Agricultural Microbiology, College of Resources and Environment, Huazhong Agricultural University, Wuhan, China; 2 Bren School of Environmental Science and Management, Earth Research Institute and University of California Center for the Environmental Implications of Nanotechnology (UC CEIN), University of California, Santa Barbara, Santa Barbara, CA, USA

**Keywords:** Nanoparticle, DNA polymerase, Metal oxides, DNA replication, Genotoxicity

## Abstract

Polymerase chain reaction (PCR) is used as an in vitro model system of DNA replication to assess the genotoxicity of nanoparticles (NPs). Prior results showed that several types of NPs inhibited PCR efficiency and increased amplicon error frequency. In this study, we examined the effects of various metal oxide NPs on inhibiting PCR, using high- vs. low-fidelity DNA polymerases; we also examined NP-induced DNA mutation bias at the single nucleotide level. The effects of seven major types of metal oxide NPs (Fe_2_O_3_, ZnO, CeO_2_, Fe_3_O_4_, Al_2_O_3_, CuO, and TiO_2_) on PCR replication via a low-fidelity DNA polymerase (Ex Taq) and a high-fidelity DNA polymerase (Phusion) were tested. The successfully amplified PCR products were subsequently sequenced using high-throughput amplicon sequencing. Using consistent proportions of NPs and DNA, we found that the effects of NPs on PCR yield differed depending on the DNA polymerase. Specifically, the efficiency of the high-fidelity DNA polymerase (Phusion) was significantly inhibited by NPs during PCR; such inhibition was not evident in reactions with Ex Taq. Amplicon sequencing showed that the overall error rate of NP-amended PCR was not significantly different from that of PCR without NPs (*p* > 0.05), and NPs did not introduce single nucleotide polymorphisms during PCR. Thus, overall, NPs inhibited PCR amplification in a DNA polymerase-specific manner, but mutations were not introduced in the process.

## Introduction

Metal oxide nanoparticles (NPs) are the most widely used engineered NPs in consumer products (Hansen et al., 2016). Besides, metal oxide nanominerals or mineral NPs are common and widely distributed in diverse environments such as soil, atmosphere, and waters (Hochella et al., 2008). These NPs can exhibit remarkable antimicrobial activity, and cytotoxicity and genotoxicity to different types of organisms (Magdolenova et al., 2014; Golbamaki et al., 2015; Mahaye et al., 2017). The proposed toxicity mechanisms of metal oxide NPs include binding of NPs to genetic material (e.g., DNA and RNA), indirect damage from NP-generated reactive oxygen species (ROS), and toxic ions released from soluble NPs (Magdolenova et al., 2014; Golbamaki et al., 2015; Peng et al., 2017). The significance of direct binding of NPs to DNA has received less attention relative to oxidative stress induced by NPs (Nel et al., 2006, 2009; Sanvicens & Marco, 2008; Kumar et al., 2011a; Peng et al., 2017). Nonetheless, prior studies showed that binding of quantum dots or Au NPs to DNA changed the normal conformation of DNA molecules (Railsback et al., 2012; Li, Zhang & Chen, 2013; Li & Chen, 2014). NPs that bind to DNA with high affinity could inhibit the normal functions of some critical DNA-binding proteins, such as RNA or DNA polymerases, by occupying protein-binding sites and impeding the movement of protein along DNA, which could result in competitive inhibition of genetic functions (McIntosh et al., 2001; Han et al., 2005; You, Chompoosor & Rotello, 2007; Johnston et al., 2010; Peng et al., 2017). Hence, the interaction between metal oxide NPs and DNA could play an important role in NP toxicity, and a complete elucidation or delineation of the underlying mechanisms is needed (Peng et al., 2017).

To study the direct physicochemical interaction between NPs and DNA, many different methods have been developed, for example, using Raman, UV-vis, and Fourier-transform infrared spectroscopy (Babu et al., 2015), isothermal calorimetric titration (Das et al., 2017), and atomic force microscopy (Li et al., 2013; Li & Chen, 2014). Besides, NP-presented polymerase chain reaction (NP-PCR) has been used to probe the effects of NPs on DNA replication. First of all, PCR experiments have shown that many NPs can inhibit DNA amplification during PCR (Li et al., 2013). For example, ZnO, CeO_2_, citrate-stabilized Au NPs, hematite NPs, citrate-stabilized Ag NPs and quantum dots at concentrations of less than one nM impeded the PCR process (Li et al., 2013). Besides, it has been shown that certain types of NPs can even promote PCR specificity and amplification efficiency. For example, colloidal Au NPs increased the sensitivity of PCR detection (Li et al., 2005a, 2005b; Mi et al., 2009; Chen et al., 2011), graphene oxide enhanced the specificity of PCR (Wang et al., 2017) and Fe_3_O_4_ NPs increased PCR yield (Kambli & Kelkar-Mane, 2016). These results indicated that the impact to PCR is varied between different types of NPs.

Nonetheless, inconsistencies were observed between NP-PCR studies. Strikingly, even studies with the same type of NPs have led to conflicting conclusions. For example, a recent study showed that DNA replication was partially inhibited by four μg/mL ZnO NPs (Khan et al., 2017), which conflicts with a previous study which demonstrated that two nM (∼1.6 × 10^−4^ μg/mL) ZnO could fully inhibit DNA replication in PCR (Li et al., 2013). Au NPs were shown to increase the efficiency of real-time PCR (RT-PCR), that utilized SuperTherm or YEA DNA polymerase, up to 10,000-fold (Li et al., 2005b), but the results could not be repeated in a recombinant Taq DNA polymerase-based PCR system (Yang et al., 2008). Also, Au NPs could reduce nonspecific products in PCR, but the effect was only observed with Taq and Tfl polymerase but not with Vent polymerase (Vu, Litvinov & Willson, 2008). The DNA templates in these studies varied, including plasmid DNA (Li et al., 2013), human cDNA (Li et al., 2005b; Mi et al., 2009), phage genomic DNA (Li et al., 2005a), and bacterial genomic DNA (Mi et al., 2009). Since different types of DNA have been shown to have different affinities for NPs (Das et al., 2017), the use of various DNA types may explain inconsistent results in assessing NP effects on PCR. Furthermore, DNA polymerase varied by study, including, for instance, Taq polymerase (Li et al., 2005a; Chen et al., 2011; Kambli & Kelkar-Mane, 2016), Ex Taq polymerase (Li et al., 2005a; Mi et al., 2009), LA Taq polymerase (Chen et al., 2011), Pfu high-fidelity DNA polymerase (Mi et al., 2009), and Phusion high-fidelity DNA polymerase (Li et al., 2013). In addition to DNA-NP interaction, the choice of DNA polymerase may also have a role in determining the results described above. To clarify this, the effects of NPs on PCR with different DNA polymerases under comparable conditions need to be studied.

In addition to compromising DNA replication, NPs may also affect the error rate in NP-PCR. Three types of Ag NPs (silver nanopowder, silver-copper nanopowder, and colloidal silver) compromised the PCR fidelity of Taq DNA polymerase (Yang et al., 2009). Further, 16 different metallic and non-metallic NPs were tested with the same polymerase, and the results showed that metallic NPs resulted in more replication errors than non-metallic NPs (Shen et al., 2009). Although many different DNA polymerases were used in NP-PCR studies, they are biologically classified into only two groups, the *Thermus aquaticus* originated A-family polymerases (e.g., Taq and Ex Taq) and the *Pyrococcus furiosus* originated B-family polymerases (e.g., Pfu and Phusion) (Ito & Braithwaite, 1991; Braithwaite & Ito, 1993). With a 3′-5′ exonuclease activity for proof reading in DNA replication, the mutation rate of B-family DNA polymerases is approximately sixfold lower than that of A-family DNA polymerases (Cline, Braman & Hogrefe, 1996). However, the two studies revealed NP effects on the replication fidelity of NP-PCR with only A-family polymerases (Shen et al., 2009; Yang et al., 2009), while the effects of NPs on the fidelity of B-family polymerases remains undetermined.

The scope of this study is, therefore, to compare the impact of metal oxide NPs on in vitro DNA replication with A and B-family DNA polymerases, and to reveal if NPs cause DNA mutations in PCR. Therefore, we tested the effects of seven types of metal oxide NPs which are either widely used in engineering or environmentally relevant as natural NPs in soils (Fe_2_O_3_, ZnO, CeO_2_, Fe_3_O_4_, Al_2_O_3_, CuO, and TiO_2_; Table 1) on low-fidelity (Ex Taq, A-family) and high-fidelity (Phusion, B-family) DNA polymerases. First, the inhibitory effects of metal oxide NPs on PCR were determined. Then, RT-PCR was used to quantify PCR efficiency. The mutation rate of PCR products was measured at a single nucleotide variation (SNV) level by high-throughput amplicon sequencing and bioinformatics analysis.

**Table 1:** Properties of nanoparticles used in this study.

Nanoparticle	Catalog #	CAS No.	Size, nm^a^	SSA, m^2^/g^a^	HDD, nm (Z-potential, mV) in nanopure water^b^	HDD, nm (Z-potential, mV) in PCR buffer^b^
Fe_2_O_3_	MKBT1848V	1309-37-1	35	50–245	878 ± 152 (9.54 ± 1.39)	941 ± 205 (−7.20 ± 0.30)
ZnO	MKBS1930V	1314-13-2	63	17	310 ± 9 (−13.13 ± 1.12)	359 ± 11 (−14.97 ± 0.42)
CeO_2_	MKBT0543V	1316-38-3	<25	N/A	179 ± 1 (6.90 ± 0.49)	528 ± 103 (−12.07 ± 0.67)
Fe_3_O_4_	MKBS1797V	1317-61-9	<100	>60	712 ± 89 (−3.89 ± 1.19)	971 ± 102 (−7.28 ± 0.85)
Al_2_O_3_	BCBK5287V	1344-28-1	<50	>40	568 ± 122 (22.67 ± 1.86)	711 ± 93 (1.31 ± 0.23)
CuO	MKBN9141V	1317-38-0	<50	29	611 ± 85 (−17.70 ± 0.78)	741 ± 51 (−14.80 ± 0.40)
TiO_2_	MKBS8073V	13463-67-7	21	51	676 ± 41 (−10.22 ± 2.10)	718 ± 38 (−5.85 ± 0.56)

**Notes:**

a The diameter of NPs reported by the manufacturer (Sigma-Aldrich).

b Measured using Zetasizer Nano ZS-90 (Malvern Instrument Ltd.) immediately after dispersing NPs in aqueous media.

SSA, specific surface area; HDD, hydrodynamic diameter; N/A, not available.

## Materials and Methods

### Nanoparticles and DNA polymerases

The NPs tested in this study were supplied from Sigma-Aldrich (St. Louis, MO, USA) (Table 1). Stock suspensions of NPs were prepared by suspending the corresponding mass of NPs in 50 mL of sterilized distilled deionized water to yield a final concentration of one mM. The size and zeta potential of the NPs in Nanopure water and in 1 × PCR buffer (20 mM Tris–HCl, pH 8.4, 50 mM KCl, two mM MgCl_2_) were measured using a Zetasizer nano ZS (Malvern, UK). DNA polymerases used in this study were either an A-family DNA polymerase (Ex-taq; Takara, Kusatsu, Japan) or a B-family DNA polymerase (Phusion; Thermo Fisher, Waltham, MA, USA).

### PCR amplification

Polymerase chain reaction was performed as previously described (Li et al., 2013). Briefly, the NP stock suspensions were sonicated (Model IID, SCIENTZ, Ningbo, China) for at least 10 min at 30 W prior to use. Then one μL of dispersed NP stock solution was added to nine μL of DNA template, mixed thoroughly using a pipette, and then incubated in an ice bath for 10 min (method adopted from Li et al. (2013)). After incubation, 10 μL of 2 × PCR master mix were added into the NP-DNA mixture and mixed thoroughly using a pipette. The amplifications were then performed in 20 μL reaction volumes with final concentrations of 1 U DNA polymerase, 50 μM NPs (except the positive controls), 200 μM each dNTP, and 0.5 μM each primer on a thermo cycler (Applied Biosystems, Foster City, CA, USA). The genomic DNA of *Bacillus subtilis* strain 168 was used as template to perform the PCR experiments. The primers used in the PCR reaction amplified a 380 bp DNA fragment of the 16S rRNA gene of *B. subtilis*. The forward primer sequence was 5′- GGGCGGTACCTTGACGGTAC-3′, and the reverse primer sequence was 5′-GGCGGAAACCCCCTAACACT -3′. The PCR product is located from 478 to 858 of the *B. subtilis* 16S rRNA gene, containing the full-length V4 region of the rRNA gene. PCR began with a denaturation step at 98 °C for 30 s, followed by 30 cycles of 30 s at 98 °C, 30 s at 60 °C, and 1 min at 72 °C. A total of 10 μL of each amplified product was used for electrophoresis using 1% agarose gel after SYBR Green I staining. The stain gel was visualized and imaged with a digital camera (Bio-Rad, Hercules, CA, USA).

### Monitoring the PCR process using real-time PCR

The RT-PCR conditions were the same as in the above-described routine PCR conditions except that the total reaction volume was limited to 10 μL and SYBR Green I fluorescence dye was added. The experiments were triplicated in a Hard-Shell 384-Well plate (Cat. hsp3805; Bio-Rad, Hercules, CA, USA) within a Flex 6 RT-PCR System (Life Technologies, Carlsbad, CA, USA). PCR began with a denaturation step at 98 °C for 30 s, followed by 40 cycles of 30 s at 98 °C, 30 s at 60 °C, and 1 min at 72 °C, and a melting curve analysis of PCR products. The fluorescence signal was collected at the end of each 72 °C step. *C_t_* thresholding was automatically determined with QuantStudio™ Real-Time Software (version 1.2).

### Amplicon sequencing

Polymerase chain reaction products were purified using the E.Z.N.A. Cycle Pure Kit (D6493-01; Omega, Biel/Bienne, Switzerland) according to the manufacturer’s instructions. DNA was dissolved in ddH_2_O and equal amounts of 10 ng DNA were then used as template for an extra PCR with barcoded primers under the same conditions, which were 95 °C for 2 min, followed by 25 cycles at 95 °C for 30 s, 55 °C for 30 s, and 72 °C for 30 s and a final extension at 72 °C for 5 min. Barcoded primers have the same complementary sequence in 3′, but in their 5′-end have an extra eight nucleotides which were unique to each library. This PCR reaction was performed in triplicate in a 20 μL mixture containing 10 μL of 2 × GC Buffer, two μL of 2.5 mM dNTPs, 0.8 μL of each primer (five μM), and 0.25 μL of Q5 DNA Polymerase (New England BioLabs, Ipswich, MA, USA). Amplicons were extracted from 2% agarose gels and purified using an AxyPrep DNA Gel Extraction Kit (Axygen Biosciences, Union City, CA, USA) according to the manufacturer’s instructions and were quantified using QuantiFluor™ -ST (Promega, Madison, WI, USA). Purified amplicons were pooled to be equimolar and paired-end sequenced (2 × 250) on an Illumina MiSeq platform according to standard protocols.

### Estimation of replication error frequency

The paired-end reads were trimmed and filtered using Trimmomatic (version 0.36) with default settings (Bolger, Lohse & Usadel, 2014). Exact barcode matching was required to de-multiplex the sequencing results of different libraries. The generated high quality reads of each library were analyzed using mothur software (version 1.40.5) (Schloss et al., 2009). Briefly, paired-read sequences were assembled using the make.contigs algorithm which aligns the sequence pairs and evaluates across the alignments for sequence overlapping to assemble paired reads into contigs. The contig counts from all sequenced libraries summed to 4.2M, with approximately 80% having a length of 380 bp which is the theoretical size of the PCR product. Each library had at least 28,457 contigs. Those contigs which had ambiguous nucleotides or had a length other than 380 bp were filtered out using the screen.seqs algorithm. The sequence error rate was estimated using the seq.error algorithm by comparing the sequencing result with the 16S rRNA gene sequence of *B. subtilis*. In addition, the error.matrix output of seq.error algorithm, which described the count of each type of SNVs as a matrix, was employed to calculate the frequency of all 12 types of SNV of each library.

### Data availability, analysis codes, and statistical analysis

The amplicon sequencing and RT-PCR raw data and analytical pipelines were deposited in GitHub (https://github.com/gaospecial/NP-PCR). Statistics were performed using R version 3.5.1 for one-way ANOVA analysis, and a post hoc test to find variations between different groups, unless otherwise stated. Normality was checked by the Shapiro–Wilk test on ANOVA residuals. If the result showed that normality is violated, ANOVA was replaced by the non-parametric Kruskal–Wallis rank sum test. In such a condition, variations between groups were tested using pairwise Wilcoxon rank sum test. The *p*-value in multiple comparisons was in certain cases adjusted by “BH” method as indicated. For all statistical analyses, a *p*-value (or adjusted *p*-value if applicable) of less than 0.05 was considered statistically significant. The GitHub repository also contains a statistics fact sheet showing these procedures.

## Results

### Reduction of PCR yield by NPs varies by polymerase family

**Figure 1:**
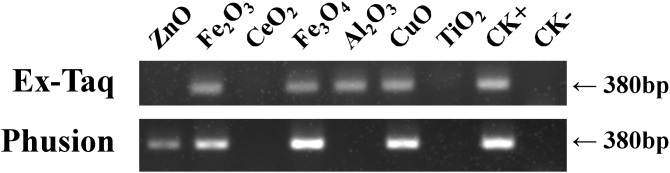
Effects of metal oxide NPs on DNA amplification by PCR as analyzed by gel electrophoresis. *B. subtilis* genomic DNA was used as a DNA template to amplify a 380 bp PCR fragment in all samples, except in the negative non-template control (CK−). Each type of NP was tested under the same final concentration (DNA incubated with 100 μM NPs and PCR conducted at a final concentration of 50 μM NPs, see methods). CK+ represents the positive control (PCR reaction without NPs). The experiments were performed in triplicate with similar results, and the representative result is shown.

Gel electrophoresis was employed to probe the effects of seven metal oxide NPs (Table 1) on the quantity of PCR amplified DNA using Ex Taq and Phusion DNA polymerases (Fig. 1). The quantity of PCR amplified DNA products in NP-containing samples was compared with the positive control (CK+), which did not contain NPs in PCR, and the negative control, which contained neither NPs nor template DNA (CK−). CeO_2_ and TiO_2_ NPs caused complete inhibition of DNA replication when using either Ex Taq or Phusion, whereas Al_2_O_3_ NPs completely inhibited DNA replication with Phusion but did not affect the DNA replication with Ex Taq. ZnO NPs completely inhibited DNA replication with Ex Taq, while DNA replication with Phusion was only partly inhibited. In contrast, other NPs did not show any signs of inhibition on the DNA replication of Ex Taq and Phusion.

Our investigation showed that PCR can be used to test the effect of metal oxide NPs on DNA replication. This is, of course, related to the dose of NP and the type of metal oxide NP (Li et al., 2013), but under the same condition of NP dosage and type, the extent to which amplification is inhibited varies with the type of DNA polymerase used during PCR. Furthermore, we used a plasmid DNA template to amplify a longer DNA fragment (1,280 bp), and found that, except the defection of Al_2_O_3_ on Phusion was less than that of Fig. 1, the experiment gave similar results (Fig. S1). The results suggested that changes on DNA template and/or target length cannot overcome the different effects of NPs on polymerases. Therefore, DNA polymerase must be considered in using the PCR method to assess the toxicity of NPs.

### Modulation of PCR efficiency by NPs

**Figure 2:**
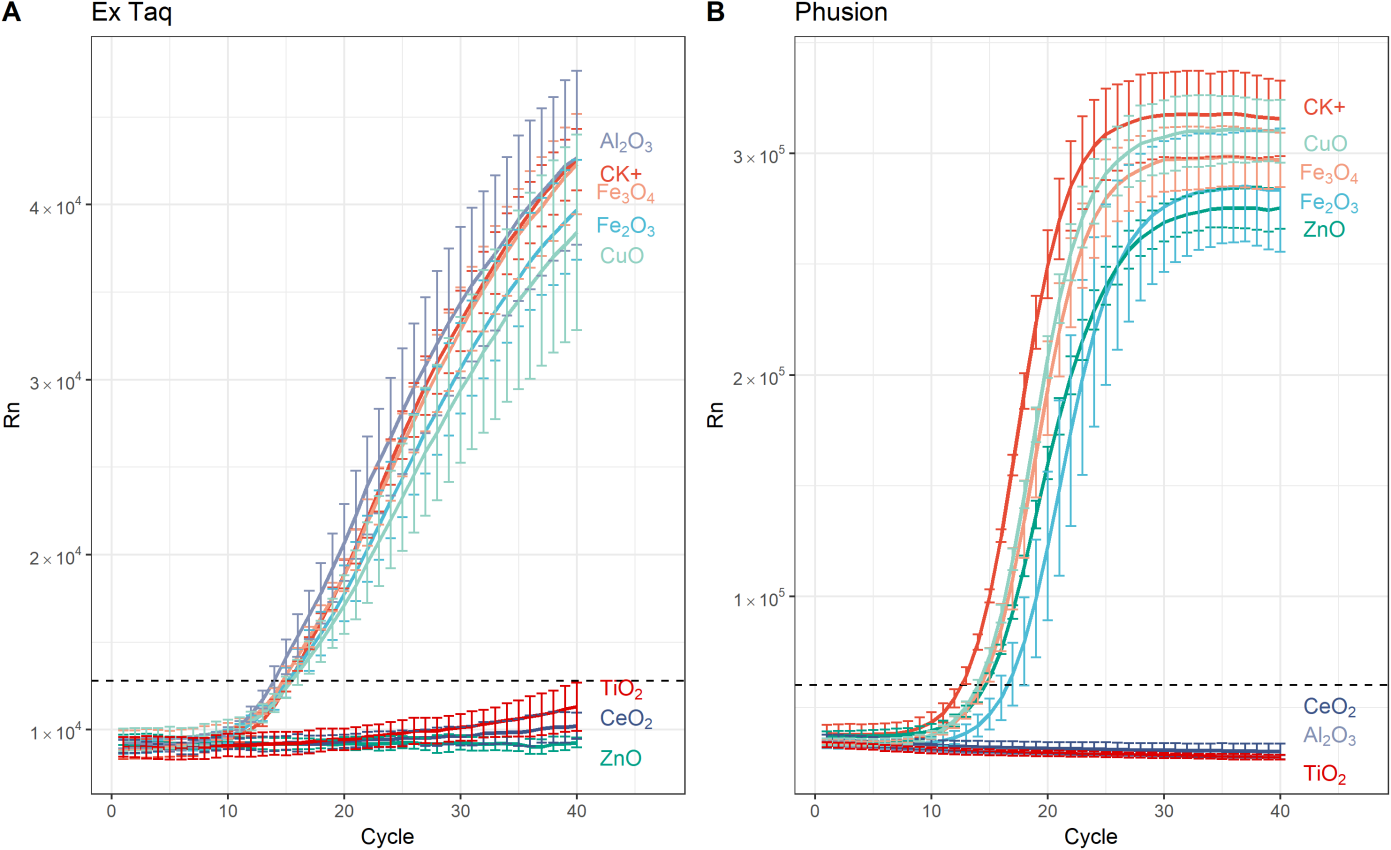
Amplification curves from real time (RT)-PCR performed using SYBR Green protocols with, or without, NPs and using either Ex Taq (A) or Phusion (B) DNA polymerase. The sample fluorescence (*y*-axis) is shown as a function of the cycle number (*x*-axis). Dashed horizontal lines indicate the cutoff for *C_t_* thresholding. For each curve, the error bars represent the standard deviation of at least three replicates. Legends on the right indicate the added metal oxide NPs or the positive control (CK+; no added NPs). The negative (no DNA template) control is not shown as there was no amplification.

**Figure 3:**
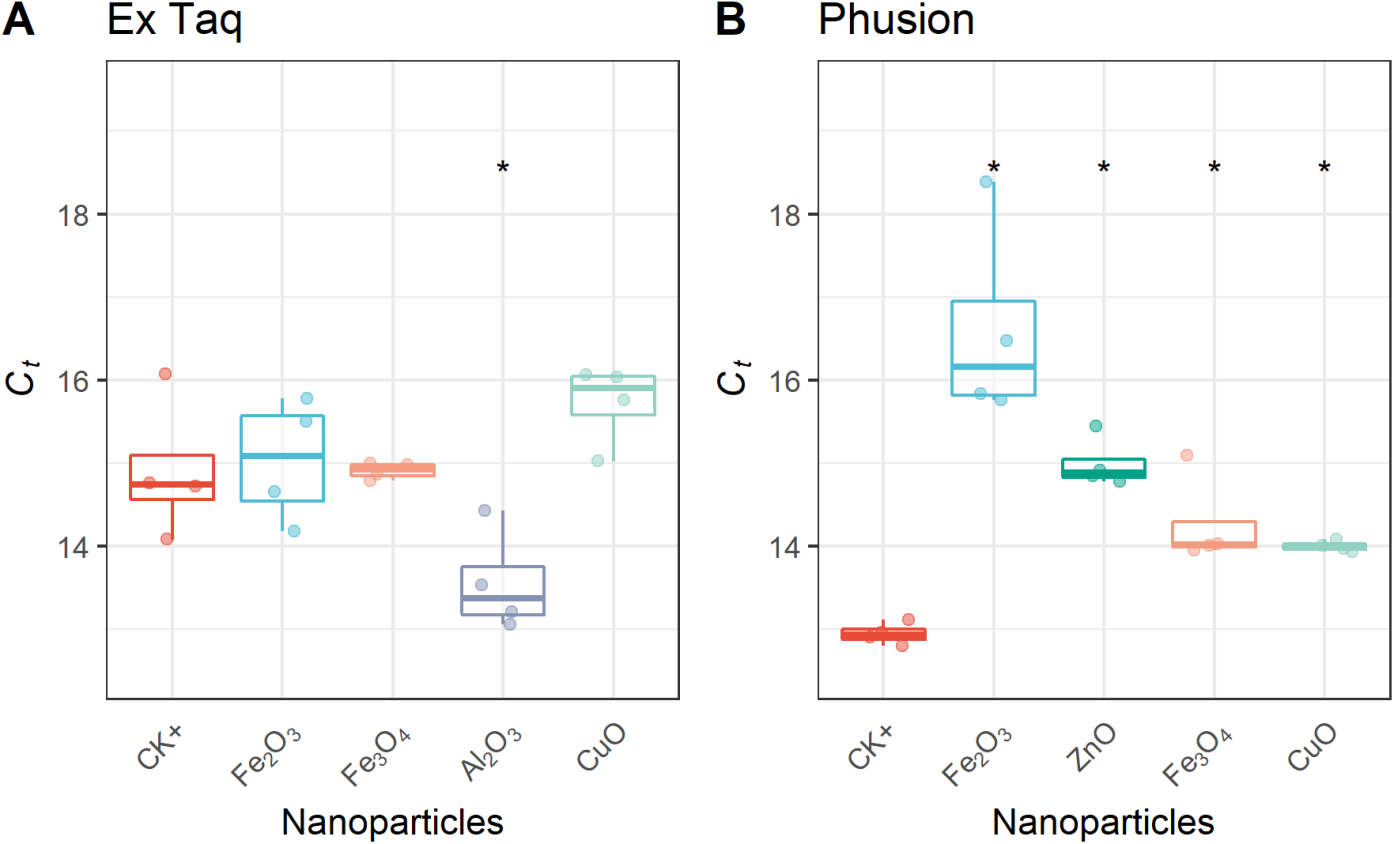
Quantitative analysis of NPs’ effects on RT-PCR efficiency. *C_t_* thresholds for NP-treated samples were compared with the positive control (CK+). The first quartile, median and third quartile are shown in the boxplots as horizontal bars, and values of each observation are shown as points within the scatter plots. For Ex Taq (A), one-way ANOVA with a post hoc analysis was performed; for Phusion (B), since the normality check failed, Kruskal–Wallis and Wilcoxon rank sum test were performed. An asterisk indicates a significant difference (*p* < 0.05) of *C_t_* values in NP-PCR sample comparing with CK+.

In addition to affecting DNA amplification by routine PCR, it has been reported that metal oxide NPs can alter the efficiency of PCR amplification (Yang et al., 2008). Therefore, we used RT-PCR to quantitatively analyze PCR efficiency in NP-amended samples. In general, RT-PCR confirmed the routine PCR results (Fig. 1), but additionally provided quantitative data on PCR yield. The complete inhibition of PCR by NPs resulted in a flat curve, indicating that some metal oxide NPs inhibited the entire PCR process (Fig. 2). In contrast, the positive control (CK+; no NP addition) and non-inhibited PCR samples yielded “S”-shape amplification curves. To quantify the effect of NPs on PCR efficiency, *C_t_* values were calculated from the “S”-shape amplification curves and statistically compared (Fig. 3). In case of reduced PCR efficiency, the *C_t_* value would increase and vice versa. When compared with the *C_t_* value of the positive control, *C_t_* values for RT-PCR reactions with Fe_2_O_3_, Fe_3_O_4_, or CuO NPs did not significantly change when Ex Taq DNA polymerase was used (Fig. 3A), indicating that these NPs did not affect Ex Taq efficiency. However, *C_t_* value of RT-PCR reactions with Al_2_O_3_ NPs were significantly lower than that of the positive control, indicating enhanced RT-PCR efficiency with Ex Taq. In contrast, when compared with the *C_t_* value of the positive control, the *C_t_* values of RT-PCR reactions with Fe_2_O_3_, ZnO, Fe_3_O_4_, and CuO NPs were significantly higher when Phusion DNA polymerase was used, indicating that the RT-PCR efficiency with Phusion was significantly reduced by these NPs (Fig. 3B).

### Replication error rate is not influenced by NPs

Metal oxide NPs could also induce toxicity by causing DNA mutations (Pan et al., 2010). In the past, the Ames test has been used to assess the extent of mutations caused by NPs (Yoshida, Kitamura & Maenosono, 2009; Pan et al., 2010; Kumar et al., 2011b; Woodruff et al., 2012; Kwon et al., 2014). With the development of sequencing technology, the potential of NPs to cause DNA mutations can be assessed by sequencing PCR amplicons. Since sequencing PCR products provides a single nucleotide resolution and does not involve cellular processes, variations in NP-induced replication errors can be distinguished from other sources of DNA damage caused by cytotoxicity (Magdolenova et al., 2014; Golbamaki et al., 2015; Peng et al., 2017; Ickrath et al., 2017).

Since each DNA polymerase confers an innate error rate, we first compared the error rate between different enzymes without the presence of NPs. The innate error rate of Phusion was found to be approximately 5.0 × 10^−4^ per base which was significantly lower (*p* = 0.0006) than that of Ex Taq at approximately 1.1 × 10^−3^ per base. This is consistent with Phusion being a high-fidelity DNA polymerase.

**Figure 4:**
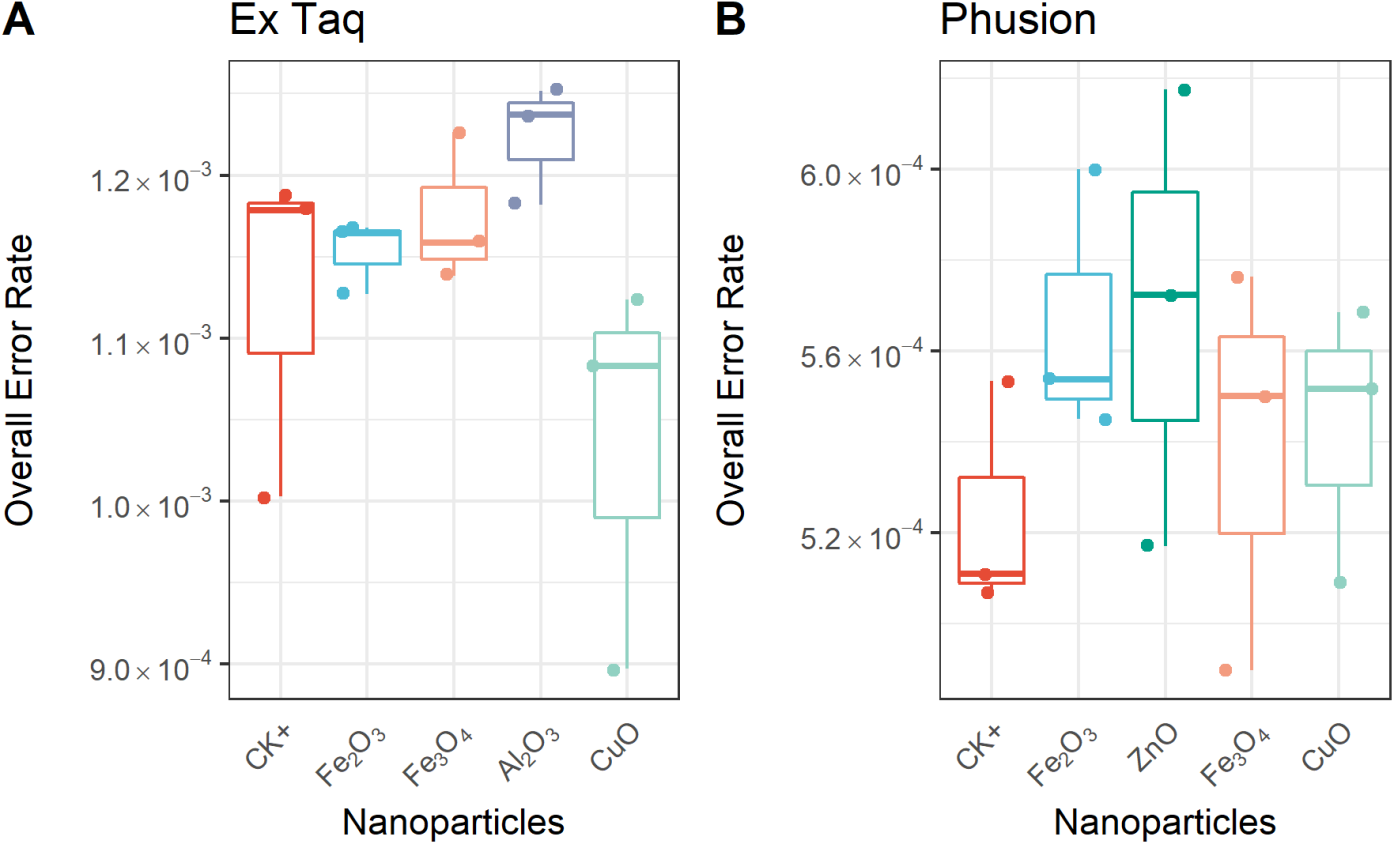
Overall error rate per base of the PCR products with or without NPs added to the PCR reaction. The frequency of single nucleotide variation (SNV) in PCR products when using either Ex Taq (A) or Phusion (B) polymerases was analyzed, as described in the Methods. The box definitions are as per Fig. 3. The differences between NP-treated PCR reactions and the positive control (CK+) were compared using ANOVA. No group showed a difference at a significance level of *p* < 0.05.

**Figure 5:**
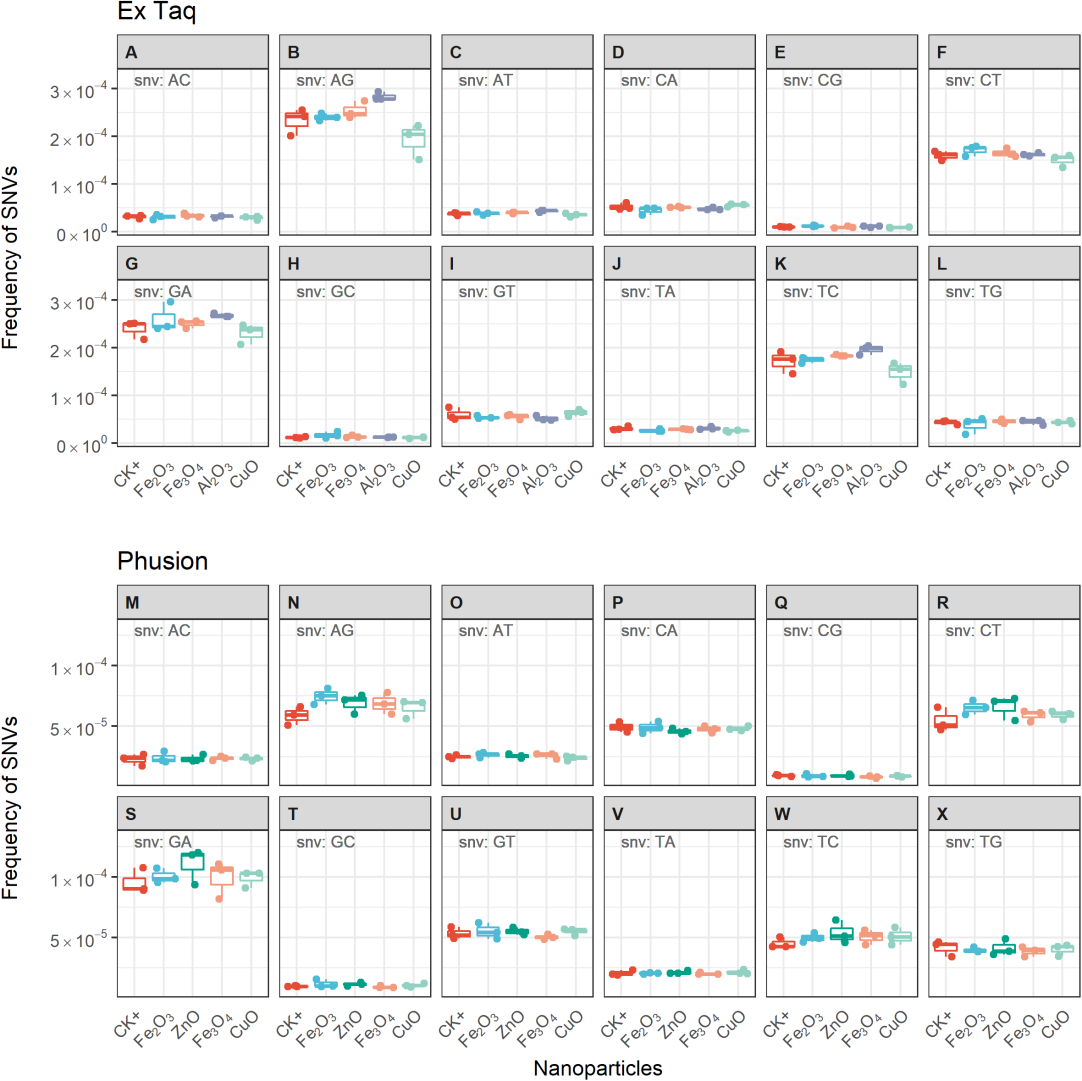
Frequencies of single nucleotide variations (SNVs) for PCR reactions using either Ex Taq (A–L) or Phusion (M–X) polymerases without (CK+) or with metal oxide NPs included in the reaction. The box definitions are the same as in Fig. 4. The insetting label on the left top of each subplot indicates the type of SNV. ANOVA was performed separately for each subplot. The *p*-values were adjusted using “BH” method. None of the adjusted *p*-values indicated significant difference between NP-treated PCR reactions and CK+ (*p* > 0.05).

We compared the error rates of amplicons from NP-treated PCR reactions with those of the positive control (CK+, no NP addition, Fig. 4). Strikingly, the tested NPs did not promote or reduce mutations in amplicons regardless of the DNA polymerase used in PCR. We furthermore compared the error rates of 12 types of SNV in PCR products, with the results indicating no significant differences between NP-treated and CK+ samples (Fig. 5). These results suggested that although the PCR efficiency was modulated by the presence of these NPs (Fig. 3), the error rate was unchanged (Figs. 4 and 5).

## Discussion

Here, we showed that the impact of NPs on PCR differed by the type of NPs and DNA polymerase. In general, the NPs capable of inhibiting PCR activity are all NPs which have been reported to have greater binding affinities to DNA, including ZnO, CeO_2_, and TiO_2_ (Li et al., 2013), suggesting that the effect of metal oxide NPs on PCR is highly dependent on the interaction between NPs and DNA. However, the PCR results with TiO_2_ NPs are different from a previous report (Li et al., 2013), and this is probably due to the high concentration used in this study, as the effect of NPs is dose-dependent (Li et al., 2013). Furthermore, different results obtained using A- and B-family DNA polymerases suggests that the activity of DNA polymerases is affected differently by metal oxide NPs in PCR reactions. This could be caused by the specificity of NP-protein interactions (Yu et al., 2017). There are two possible ways for NPs to physically interact with the DNA polymerase in PCR. One is that excess NPs that are not bound to DNA directly interact with the polymerase. The other is that the DNA which binds to the metal oxide NPs is competitively replaced by the polymerase. In addition, metal oxide NPs may release free metal ions, which may also affect the amplification efficiency of PCR (Innis & Gelfand, 1999). As a conclusion, we provide direct evidence that the effects of metal oxide NPs on DNA replication vary between different protein families associated with different polymerases.

Our results are consistent with a previous conclusion that several metal oxide NPs can inhibit RT-PCR efficiency (Shen et al., 2009). Notably, several studies have reported that NPs such as single-wall carbon nanotubes (Shen et al., 2009) and Fe_3_O_4_ (Kambli & Kelkar-Mane, 2016) can enhance PCR efficiency. However, Fe_3_O_4_ NPs only has the ability to promote PCR efficiency at concentrations much lower than those used in the above described experiments, while still inhibiting PCR at higher concentrations (Kambli & Kelkar-Mane, 2016). Therefore, the results herein, for Fe_3_O_4_ NPs, are consistent with prior reports.

Whether NPs affected DNA replication error rates was not conclusive based on previous studies (Golbamaki et al., 2015). Our results support that metal oxide NPs do not significantly increase the replication errors in DNA during PCR. It is noteworthy that Ames or other reverse mutation assays inevitably use a living cell system, which may introduce some interference. For example, NPs may induce ROS production and further cause cell DNA replication and repair process disorders (Stoimenov et al., 2002), thereby increasing the replication errors indirectly. Notably, Ames test results are inconsistent between different NPs and microbial cell types (Golbamaki et al., 2015), even in the same study (Pan et al., 2010), and sometimes rely on metabolic activation to show a change of mutation rate (Pan et al., 2010). But our results consistently show that ZnO, Fe_2_O_3_, Fe_3_O_4_, Al_2_O_3_, and CuO NPs do not cause in vitro DNA replication to be error-prone with either Ex Taq or Phusion DNA polymerases.

Based on the published literature, cytotoxicity and genotoxicity of NPs both depend on a wide range of parameters, including NP dose and physicochemical properties, such as size, surface charge, roughness, and shape (Oberdörster, 2010; Djurišić et al., 2014; Golbamaki et al., 2015). Establishing the actual effects of NPs in biological systems is a challenging task (Dhawan, Sharma & Parmar, 2009). PCR is a simple model of in vitro DNA replication that can be used for assessing the impact of NPs on DNA replication. A previous study showed that the binding affinity of NPs to DNA molecules, which can be predicted by calculating the interaction energy between NPs and DNA on the basis of DLVO models, is the most obvious reason for NPs impacting in vitro DNA replications (Li et al., 2013). We further show that metal oxide NPs vary affect the performance of DNA polymerase. Overall, most of the seven metal oxide NPs in this study have an impact on PCR, including complete inhibition of PCR and modulating PCR efficiency. Interestingly, our results indicate that the PCR efficiency associated with the high-fidelity DNA polymerase (Phusion) was significantly reduced by NPs while there was no such effect on efficiency with Ex Taq under identical experimental conditions (Fig. 3). First of all, this can explain the inconsistencies in previous studies on NP effects on PCR results—this could be because of the different polymerases used in different studies. Secondly, given that these two polymerases are derived from different organisms and belong to protein families with different biochemical activities, these results indicate that the impact of NPs are enzyme-specific and/or species-specific when affecting DNA replication. The different effects of NPs on different types of enzymes mean that if an environmental microbial community is exposed to an NP, different types of species may have differential responses. In other words, NPs can shape the community structure in environments by means of inducing varied inhibition of DNA replication, which may explain the bacterial community changes upon NP exposure (Barnes et al., 2010; Kumar, Shah & Walker, 2011; He et al., 2011).

## Conclusions

In this study, we determined whether the effects of metal oxide NPs in inhibiting two DNA polymerase PCR systems—one containing high-fidelity and another low fidelity polymerase, were different, and investigated NP-induced DNA mutant bias in different systems at the single nucleotide level. In the case of consistent doses of NPs and DNA, we found that the results of PCR differed depending on the DNA polymerase. ZnO, CeO_2_, and TiO_2_ NPs completely inhibited the DNA replication of Ex Taq, while CeO_2_, Al_2_O_3_, and TiO_2_ NPs completely inhibited that of Phusion, suggesting that the tolerance of DNA polymerase to DNA-bound NPs varied between Ex Taq and Phusion. Although ZnO, Fe_2_O_3_, Fe_3_O_4_, and CuO did not completely inhibit the DNA replication of Phusion, they significantly reduced the PCR efficiency of Phusion. By contrast, no comparable result was seen for Ex Taq, indicating that the high-fidelity DNA polymerase Phusion is more sensitive to the presence of NP. The Ex Taq PCR products with Fe_2_O_3_, Fe_3_O_4_, Al_2_O_3_, and CuO, as well as the Phusion PCR products with ZnO, Fe_2_O_3_, Fe_3_O_4_, and CuO, were subsequently sequenced, and the results showed that although the overall replication error rate of NP-presented PCR was slightly different from each other, the differences were not statistically significant when compared with normal PCR. In addition, the single nucleotide polymorphism of NP-presented PCR was not significantly changed when compared with normal PCR, either. As a conclusion, we found that typical metal oxide NPs can inhibit the DNA amplification in an enzyme-specific manner but do not significantly introduce more mutations in the process.

## Supplemental Information

10.7717/peerj.7228/supp-1 Supplemental Information 1 Supplementary Figure S1. Effects of DNA template and amplicon size in NP-PCR. (A) a hard copy of Figure 1 (for convenient comparison). (B) NP-PCR was performed with another DNA template and primers. The DNA template is pMD18T vector with a gentamycin gene insertion. The 1280 bp PCR product contains the coding sequence of the gentamycin gene and its flanking regions.

10.7717/peerj.7228/supp-2 Supplemental Information 2 The full-length uncropped gel of Figure 1. Lanes of gel were annotated as done in Figure 1 but here they show the DNA marker (M) with the DNA length on the left.

## Additional Information and Declarations

### Data Availability

The following information was supplied regarding data availability: Raw data and code are available at Github via https://github.com/gaospecial/NP-PCR.
