# Supplementary material for: Impact of metal oxide nanoparticles on in vitro DNA amplification
Source: PeerJ. 2019 Jun 27;7:e7228. doi: 10.7717/peerj.7228 (PMC6599668; doi:10.7717/peerj.7228)
Supplement: Supplemental Information 1 — (A) a hard copy of Figure 1 (for convenient comparison). (B) NP-PCR was performed with another DNA template and primers. The DNA template is pMD18T vector with a gentamycin gene insertion. The 1280 bp PCR product contains the coding sequence of the gentamycin gene and its flanking regions. [file peerj-07-7228-s001.pdf]

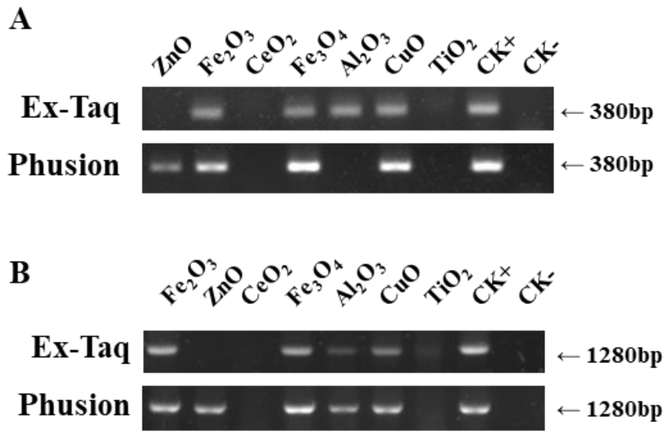

Figure S1. Effects of DNA template and amplicon size in NP-PCR. (A) a hard copy of Figure 1 (for convenient comparison). (B) NP-PCR was performed with another DNA template and primers. The DNA template is pMD18T vector with a gentamycin gene insertion. The 1280 bp PCR product contains the coding sequence of the gentamycin gene and its flanking regions.
