# Supplementary figures and images for: Impact of metal oxide nanoparticles on in vitro DNA amplification
Source: PeerJ. 2019 Jun 27;7:e7228. doi: 10.7717/peerj.7228 (PMC6599668; doi:10.7717/peerj.7228)

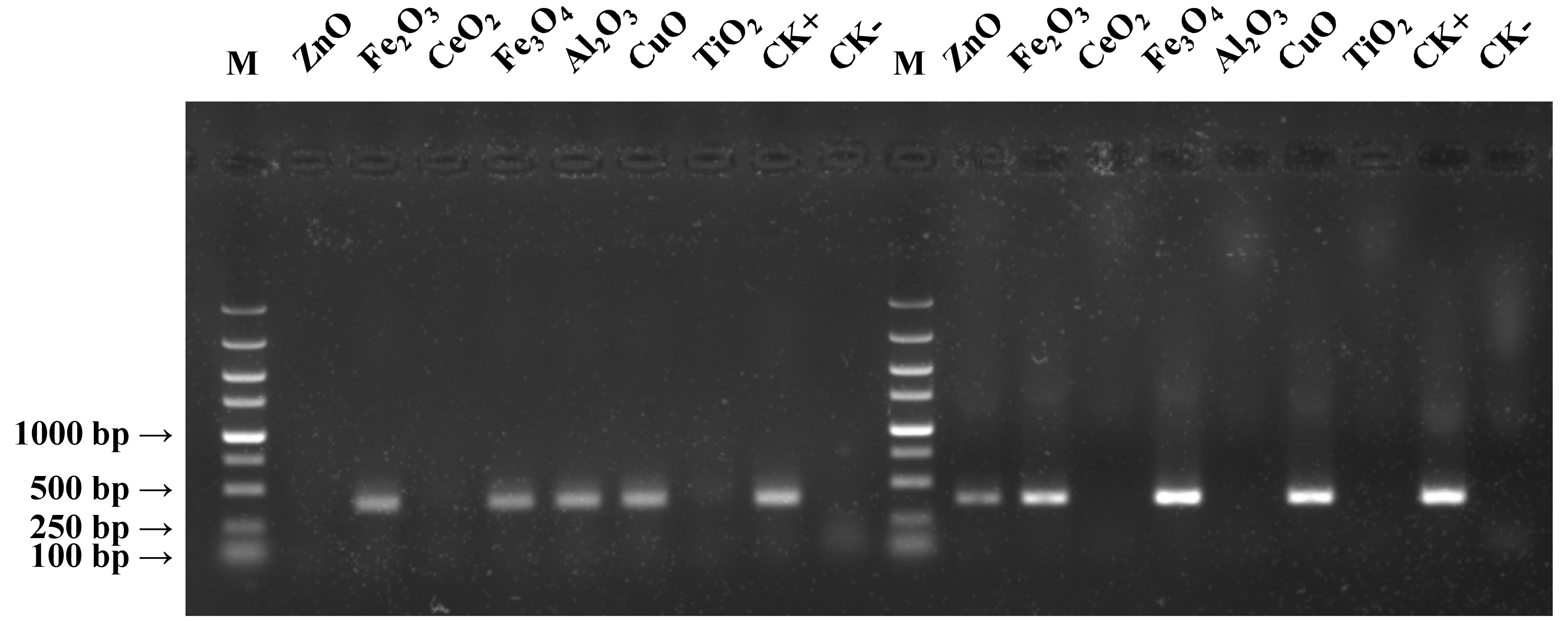

Supplement: Supplemental Information 2 — Lanes of gel were annotated as done in Figure 1 but here they show the DNA marker (M) with the DNA length on the left. [file peerj-07-7228-s002.png]
